# Identifying the Impact of Metabolic Syndrome in Hypertensive Patients

**DOI:** 10.5935/abc.20180102

**Published:** 2018-07

**Authors:** Luiz Aparecido Bortolotto

**Affiliations:** Instituto do Coração (Incor), São Paulo, SP - Brazil

**Keywords:** Metabolic Syndrome / diagnosis, Cardiovascular Diseases / mortality, Cholesterol, Triglycerides, Waist Circumference

Recent definitions of systemic arterial hypertension (AH) include metabolic changes and sustained hypertension, based on the association of AH with dyslipidemia, glucose intolerance and obesity.^1^ The mechanisms of this association are involved in the pathophysiology of AH and target-organ damage, including activation of the sympathetic nervous and the renin- angiotensin- aldosterone systems, endothelial dysfunction and inflammation.^2^ These metabolic changes, in addition to hypertension, constitute the so-called metabolic syndrome (MS), a clinical condition associated with higher risk for cardiovascular disease^3^ and chronic kidney disease.^4^ The study by Catharina et al.,^5^ published in this issue, add interesting data on the relationship between MS, repercussions of AH and resistant hypertension (RH). The study included hypertensive patients at different stages and evaluated various biomarkers, such as adipokines, as well as cardiovascular properties. The first notable finding was the high prevalence of MS in patients with AH in both RH and control groups, and the prevalence was slightly higher in the former. This result is in accordance with what has been observed in the clinical practice in the last years - a high prevalence of obesity and metabolic abnormalities associated with hypertension and its consequences.^2^ This should be considered in therapeutic approaches of these patients, and changes in life style should be encouraged aiming at better controlling blood pressure and preventing related cardiovascular diseases.^5^ The study also reported an association of MS with early kidney injury (microalbuminuria), the leptin/adiponectin ratio (L/A) and RH. No association was found between MS and increased arterial stiffness or left ventricular hypertrophy. The authors draw attention to the early detection of renal injuries in MS, regardless of the hypertension stage, emphasizing the role of metabolic changes on the development of microalbuminuria in hypertensive patients,^6^ and the need for controlling these changes to prevent kidney injury prevention. On the other hand, the lack of differences in vascular and cardiac lesions according to the presence of MS suggests that blood pressure is the component of greatest impact on these target-organ lesions, and that an adequate blood pressure control is essential for their prevention, as previously described.^7,8^ As the authors pointed out, the main finding of the study was the association between the L/A ratio and MS in hypertensive patients, reinforcing the role of increased leptin and reduced adiponectines in the pathophysiology of MS, showing as a potential therapeutic target in these patients. Besides, the L/A ratio, as suggested by the authors, may be a key tool for the screening of patients at higher risk for MS and provide them with early intervention. This, in turn, would delay the development of renal lesions and increase the likelihood of better control of blood pressure. Nevertheless, further prospective, long-term studies involving a higher number of patients are needed, as the study by Catharina et al.^5^ shows the association of the L/A ratio and MS in hypertensive patients in a cross-sectional design only. In summary, metabolic changes and obesity negatively affect blood pressure control and its repercussions in hypertensive patients and should be the target of therapeutic interventions in these individuals.

## References

[1] Malachias M, Plavnik FL, Machado CA, Malta D, Scala LC, Fuchs S (2016). 7th Brazilian Guideline of Arterial Hypertension: Chapter 1 -
Concept, Epidemiology and Primary Prevention. Arq Bras Cardiol.

[2] Kotsis V, Jordan J, Micic D, Finer N, Leitner DR, Toplak H (2018). Obesity and cardiovascular risk: a call for action from the
European Society of Hypertension Working Group of Obesity, Diabetes and the
High-risk Patient and European Association for the Study of Obesity: part A:
mechanisms of obesity induced hypertension, diabetes and dyslipidemia and
practice guidelines for treatment. J Hypertens.

[3] Kachur S, Morera R, De Schutter A, Lavie CJ (2018). Cardiovascular risk in patients with prehypertension and the
metabolic syndrome. Curr Hypertens Rep.

[4] Prasad GV (2014). Metabolic syndrome and chronic kidney disease: current status and
future directions. World J Nephrol.

[5] Catharina AS, Modolo R, Sabbatini A, Lopes HF, Moreno Junior H, Faria AP (2018). Características relacionadas à síndrome metabólica em indivíduos
com hipertensão controlada e hipertensão resistente. Arq Bras Cardiol.

[6] DeBoer MD, Filipp SL, Musani SK, Sims M, Okusa MD, Gurka M (2018). Metabolic Syndrome Severity and risk of CKD and Worsened GFR: the
Jackson Heart Study. Kidney Blood Press Res.

[7] Tedla YG, Gepner AD, Vaidya D, Colangelo L, Stein JH, Liu K (2017). Association between long-term blood pressure control and ten-year
progression in carotid arterial stiffness among hypertensive individuals:
the multiethnic study of atherosclerosis. J Hypertens.

[8] Lønnebakken MT, Izzo R, Mancusi C, Gerdts E, Losi MA, Canciello G (2017). Left ventricular hypertrophy regression during antihypertensive
treatment in an outpatient clinic (the Campania Salute
Network). J Am Heart Assoc.

